# Opening of the TAR hairpin in the HIV-1 genome causes aberrant RNA dimerization and packaging

**DOI:** 10.1186/1742-4690-9-59

**Published:** 2012-07-24

**Authors:** Atze T Das, Martine M Vrolijk, Alex Harwig, Ben Berkhout

**Affiliations:** 1 Laboratory of Experimental Virology, Department of Medical Microbiology, Center for Infection and Immunity Amsterdam (CINIMA), Academic Medical Center, University of Amsterdam, Meibergdreef 15, 1105 AZ, Amsterdam, The Netherlands

**Keywords:** HIV-1, TAR, Dimerization, Packaging, RNA structure

## Abstract

**Background:**

The TAR hairpin is present at both the 5′ and 3′ end of the HIV-1 RNA genome. The 5′ element binds the viral Tat protein and is essential for Tat-mediated activation of transcription. We recently observed that complete TAR deletion is allowed in the context of an HIV-1 variant that does not depend on this Tat-TAR axis for transcription. Mutations that open the 5′ stem-loop structure did however affect the leader RNA conformation and resulted in a severe replication defect. In this study, we set out to analyze which step of the HIV-1 replication cycle is affected by this conformational change of the leader RNA.

**Results:**

We demonstrate that opening the 5′ TAR structure through a deletion in either side of the stem region caused aberrant dimerization and reduced packaging of the unspliced viral RNA genome. In contrast, truncation of the TAR hairpin through deletions in both sides of the stem did not affect RNA dimer formation and packaging.

**Conclusions:**

These results demonstrate that, although the TAR hairpin is not essential for RNA dimerization and packaging, mutations in TAR can significantly affect these processes through misfolding of the relevant RNA signals.

## Background

**Figure 1:**
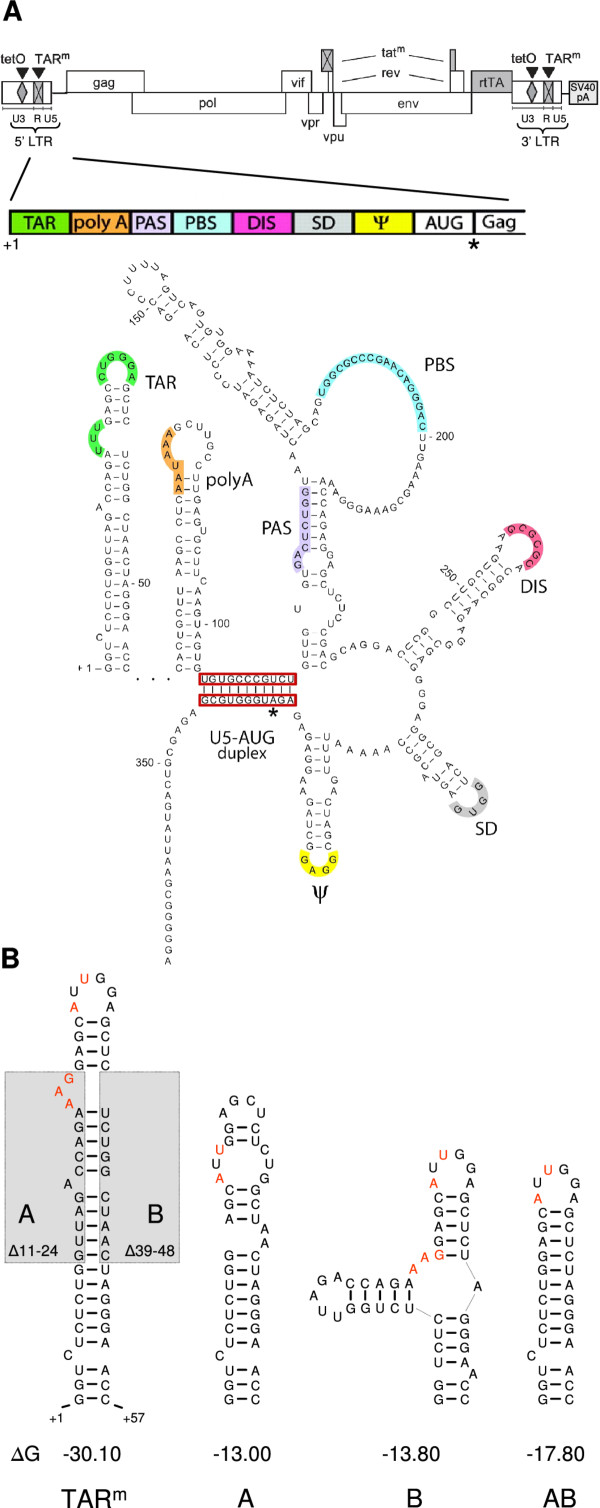
**The HIV-rtTA genome and mutations in the TAR hairpin.(A)** The HIV-rtTA proviral DNA genome and the organization and secondary structure of the leader RNA are shown. In HIV-rtTA, the Tat-TAR axis of transcription regulation was inactivated by mutation of both Tat and TAR (tat^m^ and TAR^m^; crossed boxes). Transcription and replication of the virus were made dox-dependent by the introduction of tetO elements in the U3 promoter region and replacing the Nef gene by the rtTA gene. All HIV-rtTA DNA constructs used in this study contained an SV40-derived polyadenylation signal immediately downstream of the viral genome in the plasmid backbone [17.]. The untranslated leader RNA of HIV-1 (+1/+368) can fold several stem-loop structures with important regulatory functions (TAR; polyA: polyadenylation signal; PAS: primer activation signal; PBS: primer binding site; DIS: dimerization initiation signal; SD: splice donor; Ψ: RNA packaging signal; AUG: translation start codon of gag; see text for details). **(B)** The TAR^m^ hairpin with bulge and loop mutations (in red) as present in HIV-rtTA is shown on the left side. The TAR^m^ sequence is partially deleted in the mutants A (deletion of nucleotides 11–24 [Δ11-24]), B (Δ39-48) and the double mutant AB (Δ11-24 + Δ39-48; deletions indicated by a grey box). The structure (at 37°C) and thermodynamic stability (ΔG in kcal/mole) of the TAR hairpins as predicted by the RNA Mfold program version 3.5 [18] are shown. Previous *in vitro* RNA-structure probing of leader transcripts indicated that the 3’ terminal GGGAACC nucleotides of the A and B mutated TAR elements (but not of the TAR^m^ and AB variants) interact with nucleotides immediately downstream of the polyA hairpin, which results in further destabilization of the TAR element [19.]

Human immunodeficiency virus type-1 (HIV-1) is a retrovirus with an RNA genome of approximately 9 kb that contains nine open reading frames and untranslated regions at the 5′ and 3′ end. The highly conserved leader RNA at the 5′ end contains several important regulatory RNA motifs that are involved in both early and late replication steps [1,2]. The first 97 nucleotides (nt) of this leader RNA consist of a repeat region (R) that is also present at the 3′ end of viral transcripts (Figure 1A). This repeat allows the first strand transfer step during reverse transcription and can fold into two stem-loop structures: the trans-acting responsive (TAR) element and the polyA hairpin. The 5′ TAR hairpin has an important role in transcription activation by binding the viral Tat protein and the cyclin T1 subunit of the positive transcriptional elongation factor (pTEFb) [3,4]. The polyA hairpin masks the polyadenylation signal AAUAAA, and its stability is delicately balanced to prevent premature polyadenylation at the 5′ end, yet allow efficient polyadenylation at the 3′ end [5,6]. Two important RNA elements involved in the initiation of reverse transcription, the primer binding site (PBS) and the primer activation signal (PAS), are positioned downstream of the 5′ R region in the untranslated leader (Figure 1A) [7,8]. Additional RNA signals include the dimerization initiation signal (DIS), the major splice donor site (SD) and the packaging signal Ψ. The DIS hairpin presents a 6-nt palindromic loop sequence for kissing-loop base pairing and RNA dimerization. The SD site is used for the production of all spliced transcripts, and the stability of this hairpin modulates the splicing efficiency [9]. The Ψ signal is exclusively present in unspliced transcripts and is important for packaging of the RNA genome into virions. This packaging signal is still poorly defined, and other cis-acting sequences in the HIV-1 leader RNA have been suggested to contribute to the packaging efficiency, including the upstream TAR and polyA hairpins [10-16].

*In vitro* studies demonstrated that the HIV-1 leader RNA cannot only fold the branched multiple hairpin (BMH) conformation, but also an alternative conformation in which DIS sequences interact with the polyA region [20-22]. This long-distance interaction (LDI) prevents exposure of the DIS element and can thus control the formation of RNA dimers [20,23,24]. More recently, an alternative LDI structure was proposed in which the DIS element interacts with U5 sequences downstream of the polyA hairpin [25]. This U5-DIS interaction similarly occludes the DIS loop sequence and prevents RNA dimerization. The BMH and LDI conformers may provide the virus with a riboswitch that coordinates leader RNA functions like dimerization, packaging and translation. Although the TAR hairpin is present in both leader RNA conformations, it has previously been suggested that mutations in TAR can affect the LDI-BMH riboswitch and consequently several leader RNA functions [21]. Indeed, TAR has been shown to influence dimerization of the viral RNA genome [26-28], packaging of the genomic RNA into virions [10,12-14,21,29,30] and the strand transfer step of reverse transcription [31-34]. Previous attempts to dissect the functions of TAR *in vivo*, i.e. in the context of the replicating virus, were hampered because mutations in this element cause a severe transcription and replication defect [10,29,35]. We created an HIV-1 variant with an alternative transcription axis and demonstrated that the TAR hairpin can be truncated and even completely deleted when not required for Tat-mediated activation of transcription [30]. Surprisingly, virus variants in which the TAR sequence was only partially deleted exhibited a severe replication defect. We showed that opening of the 5′ TAR element caused aberrant folding of the leader RNA *in vitro*, resulting in uncontrolled dimerization of leader RNA transcripts due to induced exposure of the DIS hairpin [19]. In this study, we used the same set of TAR mutants to demonstrate that the processes of genomic RNA dimerization and packaging in virus particles are seriously affected by misfolding of the leader RNA.

## Results

We previously designed an HIV-1 variant in which the Tat-TAR transcription mechanism was inactivated through mutation and functionally replaced by the doxycycline (dox)-inducible Tet-On gene regulation system (Figure 1A). This HIV-rtTA variant replicates exclusively in the presence of dox and does not require TAR for the activation of transcription. To study additional functions of the TAR hairpin in HIV replication, we generated HIV-rtTA variants in which the left or the right side of the TAR stem was partially deleted (A and B mutants in Figure 1B, respectively). These deletions open the TAR stem and thus destabilize this RNA element. The A and B deletions were combined in the AB double mutant, which resulted in truncation of the TAR hairpin. Whereas the A and B virus mutants showed a severe replication defect, the AB mutant replicated as efficiently as the original HIV-rtTA virus [30]. Structure probing of *in vitro* produced leader RNA transcripts demonstrated that opening of the 5′ TAR hairpin resulted in stabilization of the polyA hairpin, which forced the leader RNA in the BMH conformation [19]. We here set out to analyze which step of the HIV-1 replication cycle is affected by this mutation-induced conformational change of the leader RNA.

We first examined the effect of the TAR mutations on the production of HIV-1 RNA and its packaging into virions. C33A cervix carcinoma cells were transfected with the HIV-rtTA molecular clones that contain either the wild-type or modified TAR^m^ sequence in both LTRs (A^5+3^, B^5+3^ and AB^5+3^) and cultured with dox for two days. Because we previously observed that opening of the TAR hairpin at the 3′ end of the viral genome reduced the polyadenylation efficiency of the viral transcripts [17], all molecular clones contained an SV40-derived polyadenylation signal downstream of the viral genome. Transcripts that are not polyadenylated at the 3′ LTR site will be polyadenylated at this SV40 site, resulting in a 276-nt extension [17].

**Figure 2:**
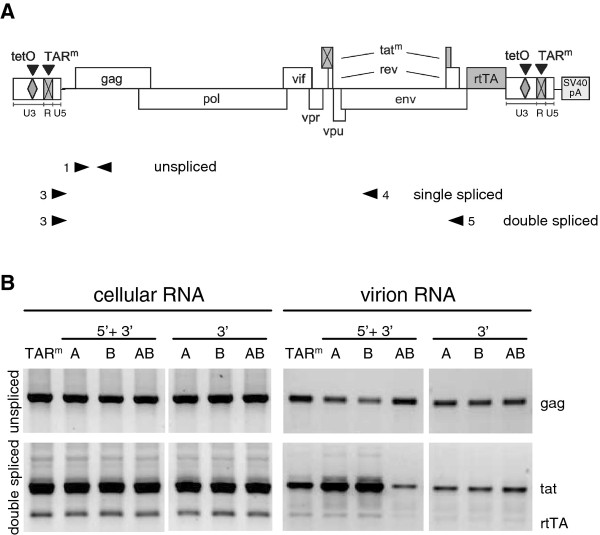
**Opening of the 5′ TAR hairpin affects packaging of viral RNAs.(A)** The position of the primers used in the RT/PCR analyses is indicated. **(B)** C33A cells were transfected with the 5′ + 3′ and 3′ mutated HIV-rtTA constructs and RNA from cells and culture supernatant was isolated after culturing with dox for 48 h. The TAR mutations did not affect virus particle production [17] and the culture supernatant samples contained similar amounts of virus particles (based on the CA-p24 level). RNA isolated from equal amounts of cells (left panels) or virus particles (right panels) was used as template for cDNA synthesis and the cDNA products were amplified with primers that specifically detect unspliced (primers 1 + 2) and double spliced transcripts (3 + 5). The identity of the PCR fragments was confirmed by sequence analysis. The band intensity corresponds with the relative amount of unspliced (upper panels) or spliced (lower panels) viral RNA present in the cells (left panels) or virions (right panels) and this assay thus allows a comparison of the level of these RNAs produced by the wild-type and TAR-mutated constructs. Because the primers, size and sequence of the PCR fragments differ for the unspliced and spliced RNAs, the band intensity of the unspliced RNA cannot be compared with that of the spliced RNAs and this RT-PCR technique does not show the absolute amount of unspliced and spliced RNAs.

To analyze the viral RNA present in cells and virions, RNA was isolated from the cells and culture medium and used for the synthesis of cDNA, which was PCR amplified with primer combinations that detect unspliced or doubly spliced viral RNAs (primers indicated in Figure 2A). As previously shown [17], the intracellular level of the unspliced and doubly spliced viral RNAs observed with the TAR-mutated constructs (A^5+3^, B^5+3^ and AB^5+3^) was similar to that observed with the wild-type HIV-rtTA construct (TAR^m^; Figure 2B, left panel), indicating that the TAR deletions do not significantly affect the production and splicing of the viral transcripts. In agreement with this, the production of virus proteins and particles (monitored by measuring the CA-p24 level in the culture supernatant) was found to be similar for the different variants (data not shown; [17]). However, marked differences were apparent when we analyzed the RNA in the extracellular virus compartment (Figure 2B, right panel). A reduced level of unspliced RNA was observed for A^5+3^ and B^5+3^ mutated viruses when compared with the wild-type virus particles. Intriguingly, the level of spliced HIV-1 transcripts was concomitantly increased in these virions. The AB^5+3^ mutated virus, which has a truncated TAR motif instead of an opened structure, did not reveal this pattern. No such effects were observed upon mutation of only the 3′ TAR element (A^3^, B^3^ and AB^3^ variants in Figure 2B). These results indicate that the reduced packaging of unspliced RNA and the increased packaging of spliced viral transcripts were due to opening of the 5’ TAR hairpin structure.

**Figure 3:**
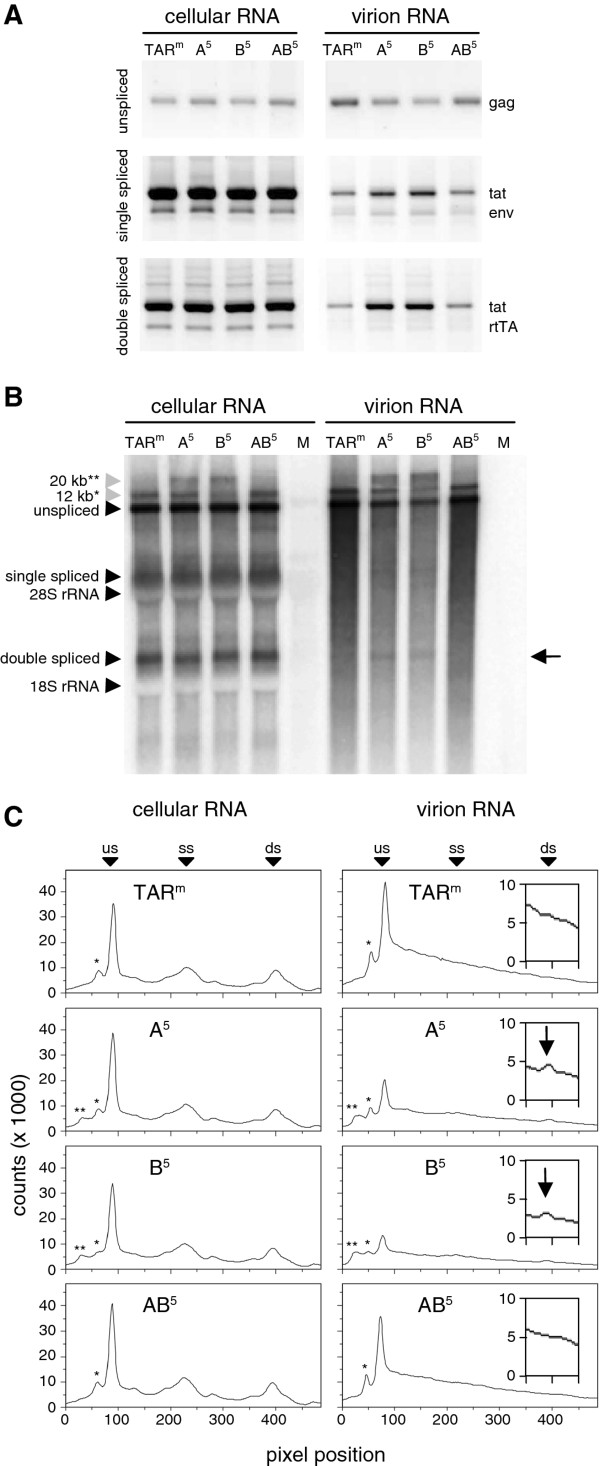
**Reduced packaging of unspliced RNAs into virions upon opening of the 5′ TAR structure.(A)** C33A cells were transfected with 5′ TAR-mutated HIV-rtTA plasmids and cultured with dox for 48 h. The TAR mutations did not affect virus protein and particle production [17] and the culture supernatant contained similar amounts of virus particles (based on the CA-p24 level). Virus particles were purified from culture supernatant samples by ultracentrifugation. Viral RNA isolated from equal amounts of cells and purified virions (based on CA-p24) was analyzed by RT/PCR using primers (as indicated in Figure 2A) that specifically detect unspliced (primers 1 + 2), single spliced (3 + 4) and double spliced transcripts (3 + 5). **(B)** The cellular and virion RNA was subsequently analyzed by denaturing Northern blotting. The position of the 18 S and 28 S rRNA bands (determined by ethidium-bromide staining; not shown), and the unspliced (9 kb), single spliced (4 kb) and double spliced (2 kb) viral transcripts are indicated. M: mock transfected cells. The arrow at the right side indicates the position of the double spliced RNAs that are visible in the A^5^ and B^5^ virion samples. *, 12-kb unspliced RNAs. As previously observed [17,], part of the viral transcripts produced from the HIV-rtTA plasmids is not polyadenylated at the 3′ LTR, which results in RNAs that are extended with vector sequences. Most of these read-through RNAs will be polyadenylated at the SV40 polyadenylation signal that was inserted immediately downstream of the 3′ LTR [17.]. However, a fraction of the transcripts is also not polyadenylated at this position, but at the downstream 5′ LTR in the circular constructs, which results in the production of 12-kb RNA fragments. In agreement with this interpretation, we previously observed that removal of the SV40 polyadenylation signal increased the 12-kb RNA level [17.]. **, 20-kb unspliced RNAs. As previously shown, the A and B mutations reduce the activity of the adjacent polyadenylation signal by stabilizing the polyA hairpin [17.]. Accordingly, the A^5^ and B^5^ mutation reduce polyadenylation of the read-through transcripts at the 5′ LTR. Subsequent polyadenylation of these read-through transcripts at the 3′ LTR or adjacent SV40 polyadenylation site can explain the production of 20-kb RNAs. These 12-kb and 20-kb RNAs form a small part of all viral RNAs, and their presence in both cells and virions is proportional to the presence of unspliced RNAs, which indicates that they are packaged with similar efficiency as the unspliced RNAs. **(C)** The profiles of the lanes from the Northern blots shown in panel B were analyzed with 1-D geI analysis software (ImageQuantTL). The position of the unspliced (us), single spliced (ss), and double spliced (ds) viral transcripts, and the 12-kb (*) and 20-kb (**) RNAs are indicated. The inserts in the right panels zoom in on the position of the double spliced RNAs, to demonstrate their presence in the A^5^ and B^5^ virion samples (indicated with arrow).

To confirm this effect of the 5′ TAR mutations on RNA packaging, we transfected C33A cells with the original and 5′-mutated HIV-rtTA variants (A^5^, B^5^ and AB^5^) and purified the virions from the culture supernatant by ultracentrifugation. RNA isolated from cells and virions was first used for cDNA/PCR analysis with primers that specifically detect unspliced and spliced transcripts (Figure 3A). The intracellular level of unspliced, single spliced and double spliced viral RNAs was similar for the wild-type and 5′ mutated constructs (Figure 3A, left panel; [17]). In agreement with this, the production of virus particles (monitored by measuring the CA-p24 level in the culture supernatant) was similar for the different variants (data not shown; [17]). The A^5^ and B^5^ mutated virions demonstrated a reduced level of unspliced RNA and an increased level of both single and double spliced RNA when compared with wild-type virions. In contrast, the RNA content of the AB^5^ virions was similar to that of wild-type virus (Figure 3A, right panel). We also analyzed the RNA isolated from cells and virions by Northern blotting (Figure 3B) and scanned the gel lanes to document differences in signal (Figure 3C). This analysis confirmed that the intracellular level of the unspliced and spliced viral RNAs was similar for the wild-type and 5′ mutated constructs (Figure 3B and C, left panels). The level of 9 kb genomic RNA in virions was also now found to be reduced for the A^5^ and B^5^ mutants, and similar to that of the wild-type virus for the AB^5^ variant (right panels). Whereas the level of the single spliced and double spliced RNA in the wild-type and AB^5^ virions was too low to detect by Northern blot analysis, the 2 kb double spliced RNAs were apparent for the A^5^ and B^5^ mutants (indicated with an arrow in Figure 3B and C). These results demonstrate that opening of the 5′ TAR hairpin structure results in reduced packaging of the unspliced RNA and increased packaging of spliced viral transcripts.

**Figure 4:**
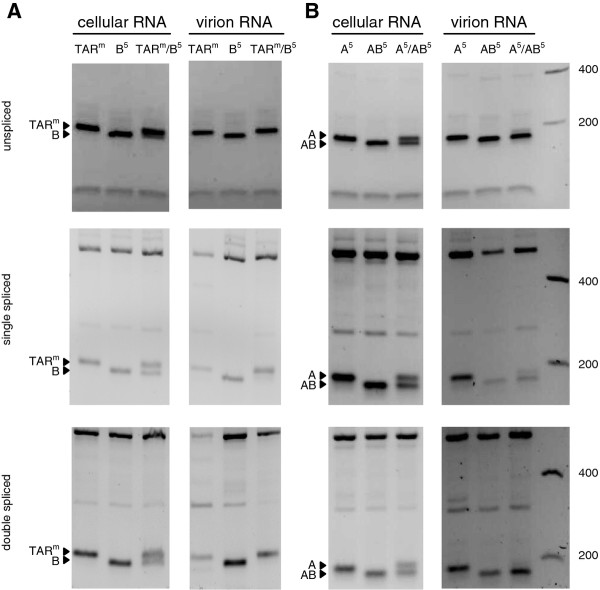
**Unspliced and spliced viral RNAs with a stable TAR hairpin are more efficiently packaged than RNAs with an opened TAR structure.(A)** C33A cells were transfected with wild-type HIV-rtTA (TAR^m^), the B^5^ mutant or both (TAR^m^/B^5^), and cultured with dox for 48 h. The intracellular and virion RNA was isolated from equal amounts of cells and culture supernatant that contained a similar amount of virus particles (based on the CA-p24 level), respectively, and used as template for cDNA synthesis. The cDNA products were amplified with primers that specifically detect unspliced (primers 1^wt/B^ and 2^+348^), single spliced (1^wt/B^ and 4) and double spliced transcripts (1^wt/B^ and 5). The PCR product was digested with NarI and the fragment containing the TAR sequence, which is 10 nt smaller for the B variant, is indicated. When necessary, the number of PCR cycles was increased to visualize all small DNA products, which obscured some of the quantitative differences between the variants as seen in Figure 3. **(B)** Viral RNAs produced upon transfection of cells with the HIV-rtTA variants A^5^, AB^5^ or A^5^ plus AB^5^ (A5/AB^5^) were analyzed as described for panel A, but PCR primer 1^wt/B^ was replaced by primer 1^A/AB^. This primer anneals to the 5′ end of the A^5^ and AB^5^ transcripts and the AB^5^ products are 10 nt smaller than the A^5^ products.

It has previously been demonstrated that spliced HIV-1 transcripts are present in virion particles, although unspliced transcripts are packaged much more efficiently [36-39]. Both type of transcripts are encapsidated through the same mechanism and compete for the same trans-acting packaging factors [39]. Accordingly, the increased packaging of spliced viral transcripts upon opening of the 5′ TAR structure may be a consequence of reduced packaging of the unspliced transcript, which would mean that TAR opening affects packaging of spliced RNA in an indirect way. Alternatively, TAR opening may affect packaging of spliced RNA in a direct way by specifically improving packaging of these spliced transcripts. To discriminate between these two possibilities, we compared the relative packaging efficiency of the wild-type (TAR^m^) and B^5^ mutated RNAs (with a stable and opened TAR hairpin structure, respectively) by co-transfecting cells with both HIV-rtTA constructs and cDNA/PCR analysis of the unspliced and spliced RNAs present in the cells and virions (TAR^m^/B^5^ lanes in Figure 4A). We used primers that anneal upstream and downstream of the 10-nt TAR deletion in B^5^, which resulted in differently sized products for the TAR^m^ and B^5^ RNAs (B^5^ products being 10 nt smaller than the TAR^m^ products). As controls, cells were transfected with only the wild-type HIV-rtTA or the B^5^ variant (TAR^m^ and B^5^ lanes). Analysis of the intracellular RNA from the TAR^m^/B^5^ transfected cells revealed a similar production of unspliced and spliced transcripts from the TAR^m^ and B^5^ constructs (Figure 4A, left panels). Analysis of the unspliced and spliced RNAs present in the virus particles resulting from this co-transfection showed that these virions contain predominantly TAR^m^ transcripts (Figure 4A, right panels). These results demonstrate that the unspliced and spliced transcripts with a stable TAR^m^ hairpin are more efficiently packaged than the corresponding B^5^ mutated RNAs with an opened TAR hairpin structure.

We similarly compared packaging of the A^5^ RNAs (with an opened TAR hairpin structure) and AB^5^ RNAs (with a truncated but stable hairpin structure) by co-transfecting cells with both HIV-rtTA mutants (A^5^/AB^5^ lanes in Figure 4B). For the cDNA/PCR analysis of the RNAs present in the cells and virions, we again used primers that resulted in differently sized products for the A and AB variants (AB products being 10 nucleotides smaller than the A products). Analysis of the intracellular RNA from the A^5^/AB^5^ transfected cells revealed a similar production of unspliced and spliced transcripts from the A^5^ and AB^5^ constructs (Figure 4B, left panels). Analysis of the unspliced and spliced RNAs in the A^5^/AB^5^ virus particles revealed the dominant presence of AB^5^ transcripts (Figure 4B, right panels). These results demonstrate that the AB^5^ mutated unspliced and spliced transcripts are more efficiently packaged than the corresponding A^5^ mutated RNAs. In both packaging competitions (TAR^m^/B and A/AB) we thus observed that opening of the 5′ TAR structure element does not only reduce the packaging efficiency of the unspliced RNA but also that of the spliced viral RNAs, which argues against a direct positive effect of TAR opening on packaging of spliced RNAs. It thus seems likely that the increased level of spliced RNAs observed in the A^5^ and B^5^ mutated virions (Figure 3) was an indirect effect of the reduced packaging of unspliced transcripts.

**Figure 5:**
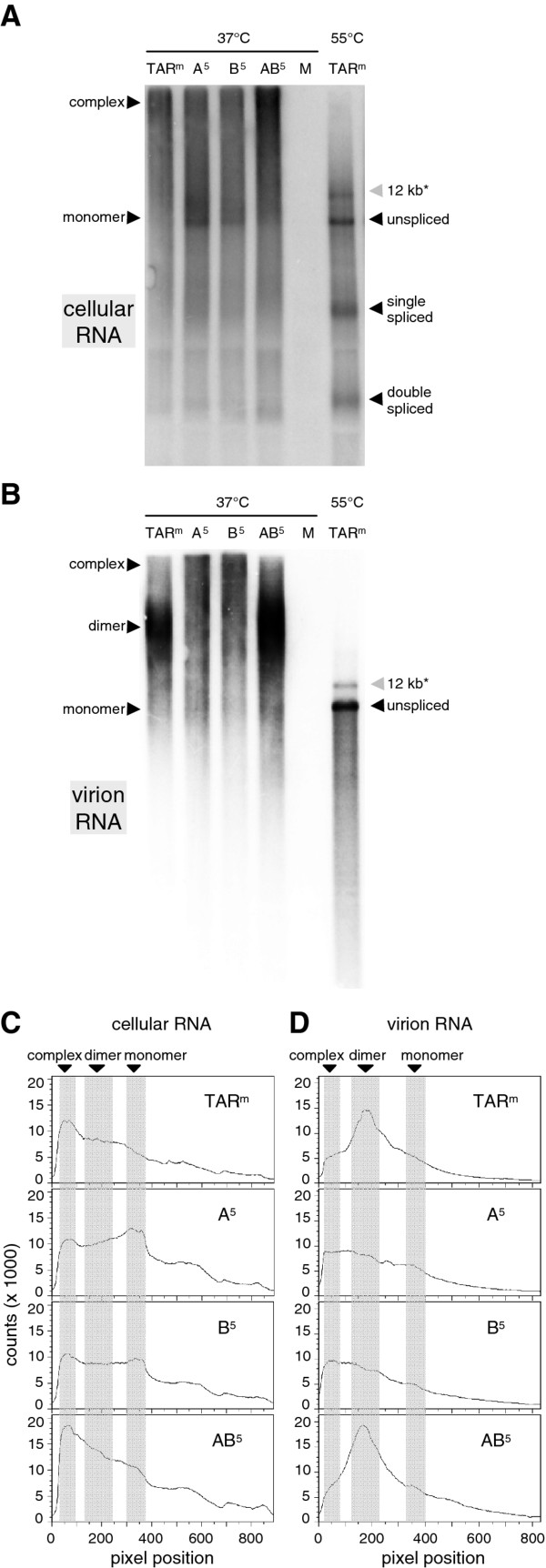
**Opening of the TAR structure affects dimerization of HIV-1 RNAs.** The cellular **(A)** and virion RNA **(B)** isolated upon transfection of cells with the original (TAR^m^) and 5′ TAR-mutated HIV-rtTA virions (as described in Figure 3) was incubated at 37°C and analyzed on a non-denaturing Northern blot. We included TAR^m^ RNA samples that were denatured by heating at 55°C before analysis to identify the position of the viral RNAs (right lanes). The position of the monomeric and dimeric unspliced RNAs, large RNA complexes, single and double spliced RNAs, and 12-kb RNAs (*, see legend to Figure 3) is indicated. M: mock transfected cells. **(C, D)** The profiles of the lanes corresponding to the non-denatured RNA samples from cells (blot shown in A) and virions (blot shown in B) were analyzed with 1-D geI analysis software (ImageQuantTL). The position of the RNA monomers, dimers and complexes are indicated.

Using *in vitro* RNA dimerization assays, we previously observed that opening of 5′ TAR (A^5^ and B^5^ mutations) caused increased dimerization of leader RNA transcripts, whereas the AB^5^ transcripts showed the wild-type pattern of regulated dimerization [19]. We therefore examined the monomeric/dimeric state of the viral RNA isolated from cells and virions by non-denaturing Northern blotting (Figure 5A and Bs). As shown in the denaturing Northern blot analysis (Figure 3B, left panel), similar levels of unspliced and spliced viral RNAs were present in the cells transfected with the original HIV-rtTA (TAR^m^) and the A^5^, B^5^ and AB^5^ variants. Analysis of these cellular samples under non-denaturing conditions revealed that the wild-type HIV-rtTA transcripts formed large intracellular complexes, which resulted in a discrete peak of RNAs with very low gel mobility (indicated with 'complex'). This HIV-rtTA sample did not show discrete RNA peaks corresponding to monomeric and dimeric 9-kb RNAs, but a smear of bands was observed at the expected positions (Figure 5A; lane profile shown in Figure 5C). The large RNA complexes were also observed for the A^5^ and B^5^ variants, but at a reduced level, while these samples demonstrated a discrete peak corresponding to 9-kb monomers (Figure 5A and C). The AB^5^ double mutation restored the wild-type RNA pattern. Comparison of the lane profiles (Figure 5C) suggests that opening the 5’ TAR structure (A^5^ and B^5^ mutations) reduces the formation of complexes, increases the level of monomers and leaves unchanged the relative level of dimers. In contrast, 5’ TAR truncation (AB^5^) did not result in such a shift from large RNA complexes to monomers. As shown in Figure 3A (right panel), denaturing Northern blot analysis of the virion RNA samples demonstrated a low genomic RNA content of the A^5^ and B^5^ virus particles, whereas the AB^5^ virions contained a wild-type level of this unspliced RNA. Analysis of these virion RNA samples under non-denaturing conditions revealed that the wild-type HIV-rtTA and AB^5^ virions contained predominantly a discrete RNA species that migrates in the gel as genomic RNA dimers, whereas the RNA from the A^5^ and B^5^ virions migrated more diffusely and did not show any discrete peak (Figure 5B and D). Taken together, these analyses indicate that opening of the 5’ TAR structure, but not truncation of this element, affects the configuration of the unspliced viral RNA in the cell and results in a reduced RNA dimer content of the virions.

## Discussion

This study is based on the surprising finding that the HIV-rtTA variant, which does not need the 5′ TAR RNA element for Tat-mediated activation of transcription, is severely replication impaired by a deletion in the left or right side of the TAR stem that opens the hairpin structure (A and B mutants), but not by the combined deletion that truncates this stem-loop element (AB). We demonstrate that introduction of the A and B deletions in the 5′ TAR element (A^5^ and B^5^ mutants) does not influence the production and splicing of viral RNA, which is in agreement with previous observations [17]. However, these TAR-opening mutations affected the configuration of the unspliced viral RNAs in cells (more monomeric RNAs, less large RNA complexes) and caused reduced packaging and dimerization of these RNAs in virions. The reduced level of unspliced RNA in virions coincided with an increased level of the spliced viral RNAs. In contrast, unspliced viral RNAs with a truncated TAR hairpin (AB^5^) exhibited the wild-type RNA pattern in cells and normal packaging and dimerization in virions. The observed changes in RNA configuration, dimerization and packaging thus correlate with the virus replication phenotype.

The unspliced HIV-1 transcript is used as mRNA for the synthesis of the Gag and Pol proteins and as genomic RNA that is encapsidated and present as dimer in the viral particle. During this packaging process, the genomic RNA must be selected from a multitude of cellular and spliced viral RNAs. This selection involves *cis*-acting RNA elements and the *trans*-acting nucleocapsid (NC) domain of the Gag protein [40]. The major packaging signal ψ is positioned downstream of the splice donor site and is thus exclusively present in the unspliced transcripts [41]. In addition to the ψ motif, other leader RNA signals upstream and downstream of the SD site have been implicated in the process of RNA packaging [2,10,13,14,16,28,39,42]. The upstream elements (including TAR, polyA, PBS and DIS) are present in both the unspliced and spliced transcripts, suggesting that these elements cannot contribute to the packaging specificity. However, although the spliced HIV RNAs are packaged much less efficiently than the unspliced RNA, they are packaged more efficiently than cellular RNAs [39], which argues for a contribution of the upstream region. Opening of the 5′ TAR structure reduced packaging of the unspliced RNA and increased packaging of spliced RNAs. These results suggest that the spliced and unspliced viral RNAs compete for packaging, which is in agreement with previous observations [36,37,39]. We demonstrated that the opened TAR structure did not selectively improve packaging of the spliced transcripts, but rather reduced the packaging efficiency of both unspliced and spliced RNAs. The relative amount of spliced transcripts increased, which indicates that packaging of the unspliced transcripts was more seriously affected.

We not only observed genomic RNA monomers and dimers when analyzing the RNA content of cells and virions, but also larger RNA complexes (Figure 5). The nature of these complexes remains unclear. Possibly, the complexes are formed through interactions between multiple unspliced viral RNAs or through interactions between the unspliced RNAs and spliced viral RNAs or cellular RNAs. We, however, cannot exclude that they are formed during or after cellular and virion RNA extraction. Whereas the unspliced RNA molecules were predominantly present as RNA dimer in TAR^m^ and AB^5^ virions, these RNAs were largely present as complexes in the corresponding cells. The A^5^ and B^5^ mutations resulted in less intracellular RNA complexes and more monomeric RNA. Whereas the intracellular level of RNA dimers was seemingly unaffected by the A^5^ and B^5^ mutations, the RNA dimer level in the corresponding virions was found to be reduced. Although it has been observed that dimerization and packaging can be dissociated [43-46], a coupling of RNA dimerization and subsequent packaging has been suggested by other studies [21,47-53]. If dimerization is a prerequisite for packaging, our results would suggest that dimeric RNA molecules with a complete or truncated 5’ TAR element (TAR^m^ and AB^5^, respectively) are more efficiently packaged in virus particles than dimeric RNA molecules with an opened 5’ TAR structure (A^5^ and B^5^). On the other hand, the direct correlation that we observe between the intracellular complex level and the RNA dimer content of the corresponding virions (TAR^m^ and AB^5^: high complex level in cells and high dimer level in virions; A^5^ and B^5^: low complex level in cells and low dimer content of virions) may suggest that the RNA complexes are the actual targets for packaging, which are later transformed into dimers. However, we have not verified the configuration of the RNA molecules in newly released virions, and it has been shown that RNA monomers (or RNAs that appear as monomers when assessed by gel electrophoresis) can also be efficiently packaged and subsequently mature into dimers [43,46].

Previous *in vitro* RNA structure probing experiments indicated that opening of the TAR structure resulted in an interaction between unpaired TAR nucleotides and downstream U5 sequences, which caused extension of the adjacent polyA hairpin [19]. Stabilization of this hairpin results in occlusion of the polyadenylation signal and reduced polyadenylation at the 3′ end of the viral transcripts [5,6,17,54]. An altered RNA conformation may also explain the deleterious effect of TAR opening in the 5′ leader RNA. Extension of the polyA hairpin with TAR and U5 nucleotides will destabilize the U5-AUG duplex that is formed immediately downstream of the polyA hairpin. It has previously been shown that such destabilization reduces dimerization of the viral RNAs [27]. Alternatively, TAR opening and subsequent polyA hairpin extension may affect dimerization more indirectly by influencing exposure of the DIS hairpin. Whereas the wild-type leader transcript preferentially adopts an LDI conformation in which the DIS signal is occluded through either a polyA-DIS or U5-DIS interaction [20-25], extension of the polyA hairpin will counteract this interaction and shift the RNA toward the alternative BMH conformation in which the DIS sequence is exposed [19]. Ooms *et al.*[21] previously showed that a change in the LDI/BMH equilibrium correlates with reduced packaging of the genomic RNA into virus particles. In agreement with this, we observed that the A^5^ and B^5^ mutations do indeed reduce packaging of the unspliced RNA. Moreover, we previously measured aberrant dimerization of TAR-mutated leader RNA molecules in in vitro assays. The A and B leader RNAs were found to dimerize more efficiently than the wild-type and AB molecules, which may be due to the increased exposure of the DIS signal in these short RNAs [19]. The current *in vivo* analysis confirms that the A^5^ and B^5^ mutations affect the dimerization process, but in this case a more complex pattern is observed. The mutations did not seemingly affect the intracellular RNA dimer level, but reduced complex formation and increased the monomeric RNA level in cells. Moreover, these TAR opening mutations reduced the RNA dimer content of the virions. These results may seem contradictory, but we note that there is good evidence that *in vitro* RNA dimerization only partially mimics the *in vivo* dimerization process. For instance, the DIS drives in vitro RNA dimerization via a loop-loop kissing interaction, but a 4-nt deletion in the loop-exposed palindrome surprisingly does not prevent HIV-1 replication and does not affect the stability of the in vivo dimer [47]. This and other results [55,56] indicate that retroviral RNA dimerization is a far more complicated process than simple DIS loop kissing, and up-regulation of what is perhaps the initial step in the RNA dimerization process may be detrimental to the complete cascade of RNA dimerization.

We previously showed that HIV-rtTA and a similarly designed dox-controlled SIV-rtTA variant replicate efficiently upon complete deletion of the TAR hairpin [30,57]. These results indicate that TAR has no essential function in the HIV and SIV life cycle other than its role in Tat-mediated activation of transcription. In agreement with this, we observed efficient packaging of HIV-rtTA genomic RNAs in which TAR was truncated (this study), completely deleted or replaced by a non-related hairpin (ER2 and ER3 variants described in [30]; results not shown). Moreover, Heng *et al*. very recently demonstrated that TAR deletion did not significantly affect RNA dimerization, NC binding and RNA packaging [58]. However, as we show in this study, opening of the 5′ TAR hairpin perturbs the leader RNA structure and affects both dimerization and packaging of the viral RNAs. Furthermore, we earlier demonstrated that opening of the 3′ TAR structure results in stabilization of the adjacent polyA hairpin that masks the polyadenylation signal, causing inhibition of polyadenylation [17]. Our combined studies thus demonstrate that although the TAR hairpin is not required for RNA packaging, dimerization and polyadenylation, mutations in TAR can affect these processes in an indirect way by disturbing the structure at the 5′ and 3′ end of the viral RNA. Apparently, the wild-type TAR hairpin is sufficiently stable to prevent detrimental interactions with other RNA domains. This insight explains the strong evolutionary pressure to close disrupted TAR hairpins in virus evolution experiments [19,30,59,60]. These studies illustrate how mutations that are designed to explore the function of a specific RNA element can affect viral replication in an indirect way through unforeseen conformational perturbation of the viral RNA genome.

## Conclusions

HIV-1 does not require TAR for dimerization and packaging of its RNA genome, but mutations in TAR can affect these processes in an indirect way. We demonstrate that opening of the TAR hairpin structure alters the leader RNA conformation, which reduces packaging and dimerization of the unspliced viral RNAs.

## Methods

### Cells and viruses

C33A cervix carcinoma cells (ATCC HTB31) [61] were grown as a monolayer in Dulbecco’s modified Eagle’s medium (DMEM) supplemented with 10% fetal calf serum (FCS) and minimal essential medium nonessential amino acids, penicillin (100 U/ml) and streptomycin (100 μg/ml) at 37°C and 5% CO_2_. Construction of the infectious HIV-rtTA molecular clone and variants with a deletion in the 5′, the 3′ or both TAR elements was described previously [30,62]. In all constructs used in this study an SV40 polyadenylation site is positioned downstream of the viral genome as described previously [17].

### RNA isolation

C33A cells were cultured to 60% confluency in 3 ml complete medium in 10-cm^2^ wells and transfected with 5 μg HIV-rtTA plasmid by calcium phosphate precipitation as previously described [62]. Cells were cultured in the presence of 1 μg/ml doxycycline (dox) (Sigma D-9891), and the culture medium was changed after 16 h. The virus level in the culture medium was quantitated at 2 days after transfection by CA-p24 enzyme-linked immunosorbent assay (ELISA) [63]. RNA was isolated from the culture supernatant by the method of Boom *et al.*[64]. The cells were washed with phosphate buffered saline (PBS), briefly incubated with 0.5 ml 0.05% trypsin-EDTA (Invitrogen) till they detached and resuspended in 1 ml 10% FCS-containing medium to inactivate trypsin. Cells were subsequently centrifuged at 2,750 g for 5 min, washed in 1 ml PBS, centrifuged at 2,750 g for 5 minutes, resuspended in 0.6 ml RLT buffer (QIAGEN) and homogenized with a QIAshredder column (QIAGEN). Total RNA was isolated with an RNeasy kit (QIAGEN) and contaminating DNA was removed with RNase-free DNase (QIAGEN) during isolation.

For the isolation of RNA from purified virions, C33A cells were cultured in 20 ml complete medium in 75-cm^2^ plates to 60% confluency, transfected with 40 μg HIV-rtTA and cultured with 1 μg/ml dox. The culture supernatant was harvested 2 days after transfection and cells were removed by low-speed centrifugation (10 min at 1500 rpm). The supernatant was filtered through a 0.45-μm filter and virion particles were pelleted by centrifugation at 32,000 rpm (175,000 g) for 90 min at 4°C in a Beckman SW32 Ti rotor. The virions were resuspended in 0.6 ml RLT buffer. The RNA was isolated as described above and resuspended in 50 μl water.

### Northern blot analysis

For denaturing Northern blot analysis, 5 μg cell-derived RNA in 10 μl water or 10 μl virion-derived RNA were mixed with 10 μl denaturing sample buffer (80 mM MOPS pH 7.0, 20 mM sodium acetate, 14% formaldehyde, 0.1 mg/ml ethidium bromide, 1 mg/ml orange G, 13 g/ml sucrose), heated at 65°C for 10 min and subsequently electrophoresed on a 1% agarose gel in MOPS buffer (40 mM MOPS, 10 mM sodium acetate, pH 7.0) with 7% formaldehyde at 100 V for 4 h. For non-denaturing Northern blot analysis, 10 μl RNA was mixed with 5 μl sample buffer (30% glycerol and 0.25% bromophenol blue), incubated for 10 min at 37°C (or 55°C when indicated) and electrophoresed on a 0.9% agarose gel in 1 x Tris-Borate-EDTA buffer at 100 V for 4 h. The RNA was subsequently denatured by soaking the gel in 3 volumes of 10% formaldehyde at 65°C for 30 min.

The RNA was transferred onto a positively charged nylon membrane (Boehringer Mannheim) with 20 x SSC by means of capillary force for 16 h. The RNA was linked to the membrane using a UV crosslinker (Stratagene). A ^32^P-labeled probe corresponding to the nearly complete HIV-rtTA genome was generated by random-primed labeling (High Prime DNA Labeling kit; Roche Diagnostics) of the 3.9 and 5.5 kb SalI fragments derived from the HIV-rtTA plasmid. Prehybridization and hybridization was done in ULTRAhyb buffer (Ambion) at 55°C for 1 and 16 hours, respectively. The membrane was then washed two times at room temperature for 5 min with low-stringency buffer (2 × SSC, 0.2% SDS) and two times for 10 min at 50°C in high stringency buffer (0.1 × SSC, 0.2% SDS). Images were obtained using the PhosphorImager (Amersham Biosciences) and data analysis was performed with the ImageQuant software package.

### RT/PCR analysis of viral RNA

RNA was reverse transcribed with ThermoScript reverse transcriptase at 50°C (Invitrogen) using the supplied oligo(dT)_25_ and random hexamers primers. The cDNA product was used as template in a PCR assay with primers 1 (GAG ACC ATC AAT GAG GAA GCT GCA GAA TGG GAT; position +942 /+974 with +1 as the transcription start site) and 2 (GGC CGG CCC TTG TAG GCC GGC CAG ATC TTC CC; +1663/+1638) to detect unspliced RNA, with primers 3 (TCA ATA AAG CTT GCC TTG AGT GC; +71/+93) and 4 (CTA TGA TTA CTA TGG ACC ACA CA; +5724/+5702) to detect the single spliced RNA, and with primers 3 and 5 (CTC CGC AGA TCG TCC CAG AT; +8102/+8083) to detect the double spliced RNA. The cDNA was denatured at 94°C for 5 min and PCR-amplified in 30 cycles of 1 minutes 95°C, 1 minutes 55°C, 2 minutes 72°C and a final extension time of 7 minutes at 72°C. The PCR products were visualized on a 1% agarose gel stained with ethidium bromide. In the co-transfection experiment, the unspliced transcript was detected with primers 1^wt/B^ (TAA TAC GAC TCA CTA TAG GTC TCT CTG GTT AGA CCA G; HIV-rtTA positions +1/+20 underlined) or 1^A/AB^ (CTA ATA CGA CTC AGT ATA GGG TCT CTC TG-Δ11/24-GAGC ATT GGA; HIV-rtTA positions +1/+34 underlined) plus 2^+348^ (CAT CGA TCT AAT TCT CCC CCG CTT AAT ACT GAC GC; +382/+348), and the splice products were detected with primers 1^wt/B^ or 1^A/AB^ plus 4 (single spliced) or 5 (double spliced). The PCR products were digested with NarI before gel analysis.

## Competing interests

The authors declare that they have no competing interests.

## Authors’ contributions

ATD designed research, analyzed data and wrote manuscript. MMV designed and performed experiments and wrote manuscript. AH performed experiments and edited manuscript. BB designed research and wrote manuscript. All authors read and approved the final manuscript.
